# Global and China burden and trend prediction of thyroid cancer attributed to high BMI from 1990 to 2021

**DOI:** 10.3389/fonc.2025.1670286

**Published:** 2026-01-13

**Authors:** Zhibo Teng, Yangfan Dong, Shaobin Duan

**Affiliations:** 1 The Fourth Affiliated Hospital of Xinjiang Medical University, Urumqi, China; 2 The Fourth Clinical School of Xinjiang Medical University, Urumqi, China

**Keywords:** age-period-cohort model, disease burden, high BMI, prediction, thyroid cancer

## Abstract

**Background:**

With economic development and lifestyle changes, the prevalence of obesity has increased significantly. However, limited research has investigated the trends in the burden of thyroid cancer attributable to high BMI.

**Methods:**

This study utilized data from the Global Burden of Disease (GBD 2021) study. Joinpoint regression analysis was used to compute the average annual percentage change (AAPC) in age-standardized disability-adjusted life year (DALY) rates (ASDR) and age-standardized mortality rates (ASMR). An age-period-cohort (APC) model was used to analyze the effects of age, period, and birth cohort on the burden of thyroid cancer attributable to high BMI. The ARIMA model was applied to predict the global and national disease burden from 2022 to 2031.

**Results:**

In 2021, the rates of change in DALY number, crude DALY rate, ASDR, death number, and crude death rate for thyroid cancer attributable to high BMI in China exceeded the corresponding global rates compared to 1990. From 1990 to 2021, the global burden of thyroid cancer attributable to high BMI was higher in women than in men, whereas in China, the burden was higher in men than in women from 2010 to 2021. The peak burden occurred in middle-aged populations, with the burden increasing with age. From 1990 to 2021, the ASDR and ASMR for thyroid cancer attributable to high BMI showed an upward trend globally and in China. Projections indicate that the burden will continue to rise from 2022 to 2031.

**Conclusion:**

From 1990 to 2021, the ASDR and ASMR for thyroid cancer attributable to high BMI exhibited an increasing trend globally and in China. The burden was higher in women than in men globally, but in China, it was higher in men than in women from 2010 to 2021. The burden increased with age. Projections suggest that the burden of thyroid cancer attributable to high BMI will continue to rise from 2022 to 2031.

## 1 Introduction

Cancer remains a major global public health challenge, with hormone-related cancers garnering significant attention due to their distinct pathogenesis and epidemiological characteristics (1, 2). These cancers include breast, thyroid, endometrial, ovarian, prostate, and testicular cancers, all of which are influenced by endogenous or exogenous hormonal regulation. With global aging and lifestyle changes, the incidence rate of thyroid cancer has been rising (3–5), ranking as the ninth most common cancer worldwide and posing a significant public health problem (6). Thyroid cancer originates from malignant tumors of thyroid follicular epithelial or parafollicular cells and is the most common endocrine malignancy (7), with approximately 90% being differentiated thyroid cancers (8). The incidence and mortality of thyroid cancer have undergone significant changes, profoundly impacting patients’ overall health and quality of life (9).

High body mass index (BMI) is a lifestyle factor closely associated with premature mortality. Expert consensus and guidelines indicate (10–15) that obesity is a modifiable metabolic risk factor influenced by genetic, environmental, socioeconomic, and behavioral factors (16). Studies have shown that obesity is a risk factor for many chronic diseases, including hypertension (17), diabetes (18), cardiovascular diseases (19), and liver cirrhosis (20). Research published in *The Lancet* found that obesity also increases the risk of cancer (21). In 2020, the global healthcare cost of obesity approached $ 1.96 trillion, accounting for 2.4% of global GDP (16, 22). By 2035, the economic impact of obesity is projected to reach nearly 3% of global GDP (23). Obesity not only affects public health but also imposes a heavy burden on families, society, and healthcare systems (24, 25).

Although high BMI is a modifiable behavioral risk factor, it has not received sufficient attention in oncology, particularly regarding thyroid cancer. Studies have found that obesity and overweight are significant risk factors for the onset and progression of thyroid cancer (26–28). Schmid et al. (29) reported that for every 5-unit increase in BMI, 5 kg increase in weight, 5 cm increase in waist or hip circumference, or 0.1-unit increase in waist-to-hip ratio, the risk of thyroid cancer increases by 30%, 5%, 5%, and 14%, respectively. MASONE S et al. (30) found that immune cells residing in fat cells enhance the production of pro-inflammatory factors, which are associated with oxidative stress and cancer development or progression.

Over the past decade, economic growth, urbanization, dietary changes, and physical inactivity have led to a sharp rise in obesity prevalence (31, 32). Nearly a quarter of adults fail to meet the World Health Organization (WHO) recommendations for physical activity (33). In 2015, 603.7 million people worldwide were obese (34), and studies predict that obesity prevalence will continue to rise (35). Research by Wang Limin et al. (36) published in *The Lancet* showed that the prevalence of obesity (BMI ≥ 30 kg/m²) in China increased from 3.1% to 8.1%. In 2018, an estimated 85 million adults aged 18–69 in China were obese (48 million men and 37 million women), more than triple the number in 2004 (28 million). Reducing BMI is one of the most cost-effective ways to lower the risk of multiple diseases (37). On March 9, 2025, China launched a three-year “Weight Management Year” initiative to promote comprehensive weight control and BMI reduction. Therefore, monitoring high BMI prevalence is crucial for developing and implementing public health programs aimed at mitigating or eliminating the impact of obesity. However, there is relatively little research on the burden of thyroid cancer attributable to high BMI in China and its trends across genders and age groups. This study, based on the Global Burden of Disease (GBD 2021) database, analyzes the current status and trends in the burden of thyroid cancer attributable to high BMI globally and in China from 1990 to 2021, providing a scientific basis for policies to prevent and control overweight/obesity.

## 2 Materials and methods

### 2.1 Data sources

The GBD 2021 database covers 204 countries and regions, 371 diseases, and 87 risk factors. Using the GBD 2021 database (38), data on the burden of thyroid cancer attributable to high BMI globally and in China from 1990 to 2021 were obtained through the online GHDx query tool (*http://ghdx.healthdata.org/gbd-results-tool*). The search criteria included: GBD Estimate (Risk factor), Measure (DALYs & Deaths), Metric (Number & Rate), Risk (High body-mass index), Cause (Thyroid cancer), Location (Global & China), Age (All), Sex (All), and Year (All). Indicators of the burden of thyroid cancer attributable to high BMI included DALYs, deaths, and their 95% uncertainty intervals (UI), as well as corresponding crude rates (DALY rate, death rate) and age-standardized rates (ASDR, ASMR). Age standardization was performed using the GBD 2021 global standard population structure. This study complies with the Declaration of Helsinki (2013 revision) and does not involve data that could harm patient privacy or disclose personal identities. The use of GBD data adheres to all relevant legal and ethical guidelines, as well as the Creative Commons Attribution-NonCommercial-NoDerivatives 4.0 International License and the University of Washington’s terms and conditions (39).

### 2.2 Definition of thyroid cancer attributable to high BMI

Thyroid cancer was defined as a malignant tumor originating from thyroid follicular epithelial or parafollicular cells, further classified into papillary, follicular, poorly differentiated, anaplastic, and medullary carcinomas. Diagnostic codes for thyroid cancer included ICD-10 codes C73-C73.9, D09.3, D09.8, D34-D34.9, D44.0, and Z85.850, and ICD-9 codes 193-193.9 and 226-226.9 (7, 39, 40). High BMI was defined as BMI ≥ 25 kg/m² for adults (aged 20+) (41–45).

### 2.3 Statistical methods

R 4.30 was used to calculate the change rates in DALY and mortality rates. Joinpoint 5.02 software was employed to compute the average annual percentage change (AAPC) and annual percentage change (APC) with 95% confidence intervals (CI) for each time segment. The joinpoint regression model was specified as: ln(ASDR) = *α* + β_i_x + *ϵ*. APC = 100 × [exp(β_i_)-1], AAPC = [∑(w_i_ × APC_i_)]/(∑w_i_); where *x* represents calendar year, *β*_i_ represents the slope coefficient for each identified segment, *w*_i_ is the segment-specific time span weight, A statistically significant increasing trend in ASDR was concluded when the lower bound of the AAPC’s a lower 95% confidence limit > 0. Conversely, a significant decreasing trend was identified when the upper 95% confidence limit < 0. Otherwise, the ASDR was considered stable (46).

The age-period-cohort (APC) model was used to assess the effects of age, period, and birth cohort on outcomes. The APC analysis was conducted using the online APC Web Tool (*http://analysistools.nci.nih.gov/apc/*) provided by the National Cancer Institute. This tool is supported by built-in estimable function algorithms and corresponding Wald tests (47). The intrinsic estimator algorithm and Wald tests were used for parameter hypothesis testing. Age groups were divided into 16 segments (20–24 to 95+ years) at 5-year intervals. The study period spanned from 1992 to 2021, divided into six 5-year intervals, with median years (1994, 1999, 2004, 2009, 2014, 2019) representing each interval. Birth cohorts were derived by subtracting age from period and divided into 21 cohorts (1897–1997). The age effect represents the biological association between aging and the burden of thyroid cancer attributable to high BMI. The period effect captures temporal changes in the risk of thyroid cancer attributable to high BMI across calendar years. The cohort effect reflects generational societal changes in susceptibility to thyroid cancer attributable to high BMI (48).

The autoregressive integrated moving average (ARIMA) model was used to predict the global and national burden of thyroid cancer attributable to high BMI from 2022 to 2031. The Ljung-Box test was applied to evaluate the model, and the optimal model was selected based on the smallest Bayesian information criterion (BIC) and root mean square error (RMSE). The significance level was set at (α = 0.05).

## 3 Results

### 3.1 Global and national burden of thyroid cancer attributable to high BMI

In 2021, the global DALYs and deaths due to thyroid cancer attributable to high BMI were 144,955 and 5,255, respectively, representing increases of 134.50% and 139.08% compared to 1990. The crude DALY and death rates in 2021 were 1.84 and 0.07 per 100,000, respectively, increases of 58.62% and 75.00% from 1990. The global ASDR in 2021 was 1.68 per 100,000, a 12.75% increase from 1990 (**Table 1**).

**Table 1:** Burden of thyroid cancer attributable to high BMI globally and in China in 1990 and 2021.

Year	DALYs number	Cude DALY rate(per 100,000)	ASDR(per 100,000)	Deaths number	Cude death rate(per 100,000)	ASMR(per 100,000)
Global						
1990(n, 95%%UI)	61815 (4.6571, 79116)	1.16 (0.87, 1.48)	1.49 (1.12, 1.9)	2198 (1642, 2818)	0.04 (0.03, 0.05)	0.06 (0.04, 0.07)
2021(n, 95%%UI)	144955 (109230, 184747)	1.84 (1.38, 2.34)	1.68 (1.26, 2.14)	5255 (3914, 6653)	0.07 (0.05, 0.08)	0.06 (0.05, 0.08)
China						
1990(n, 95%%UI)	8267 (5660, 11487)	0.7 (0.48, 0.98)	0.91 (0.62, 1.26)	275 (187, 380)	0.02 (0.02, 0.03)	0.04 (0.02, 0.05)
2021(n, 95%%UI)	23684 (16056, 32507)	1.66 (1.13, 2.28)	1.15 (0.78, 1.57)	871 (588, 1177)	0.06 (0.04, 0.08)	0.04 (0.03, 0.06)

DALYs, disability-adjusted life-years; n, number; UI, uncertainty interval; ASDR: age-standardized DALY rate; ASMR: age-standardized mortality rate.

In China, the DALYs and deaths in 2021 were 23,684 and 871, respectively, increases of 186.49% and 216.73% compared to 1990. The crude DALY and death rates in 2021 were 1.66 and 0.06 per 100,000, increases of 137.14% and 200.00% from 1990. The ASDR in 2021 was 1.15 per 100,000, a 26.37% increase from 1990 (**Table 1**).

**Figure 1:**
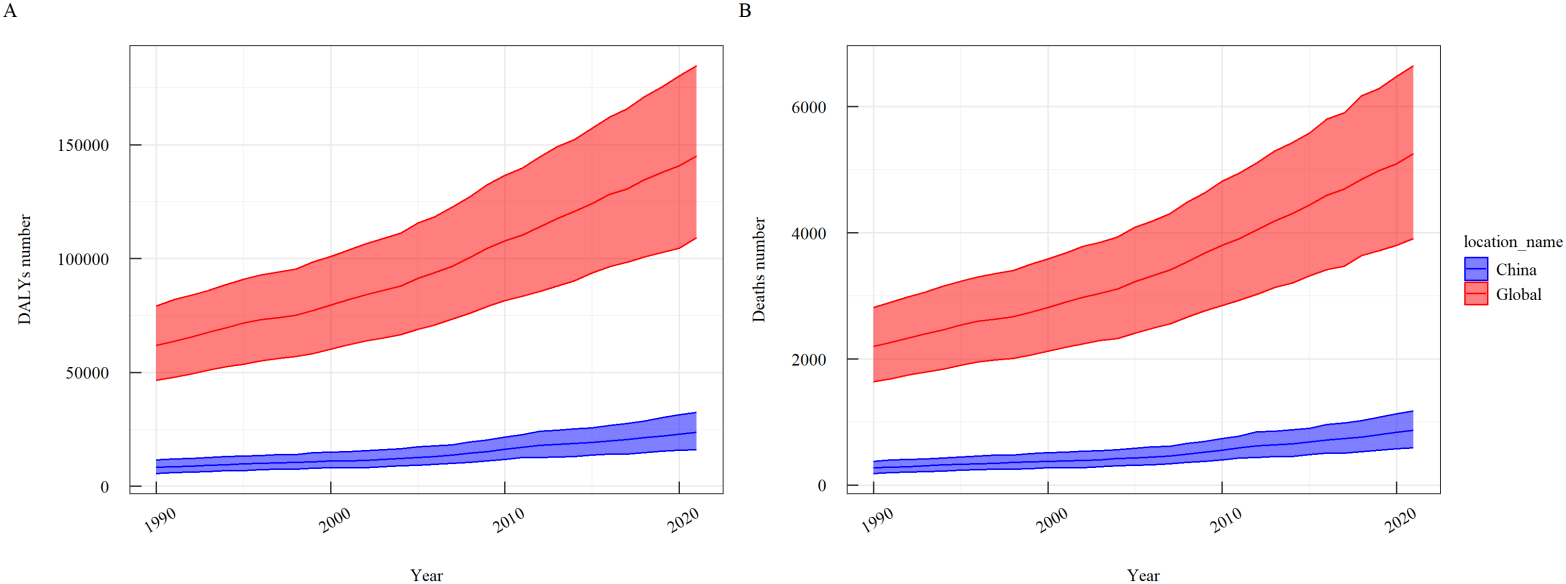
Trends in the burden of thyroid cancer attributable to high BMI globally and in China, 1990–2021. **(A)** DALY Number; **(B)** Death Number.

The change rates in DALY number, crude DALY rate, ASDR, deaths number, and crude death rate in China were higher than the global rates. From 1990 to 2021, the global and national DALYs and deaths due to thyroid cancer attributable to high BMI showed an overall upward trend (**Figure 1**).

### 3.2 Gender and age-specific burden of thyroid cancer attributable to high BMI

In 2021, the global DALYs and deaths among men were 56,835 and 2,029, respectively, increases of 172.21% and 188.21% from 1990. The crude DALY and death rates in 2021 were 1.44 and 0.05 per 100,000, increases of 84.62% and 66.67% from 1990. The ASDR and ASMR in 2021 were 1.38 and 0.05 per 100,000, increases of 30.19% and 25.00% from 1990 (**Table 2**).

**Table 2:** Gender-specific burden of thyroid cancer attributable to high BMI globally and in China in 1990 and 2021.

Variable	DALYs number	Cude DALY rate(per 100,000)	ASDR (per 100,000)	Deaths number	Cude death rate(per 100,000)	ASMR(per 100,000)
Global Male						
1990(n, 95%%UI)	20879 (15682, 26681)	0.78 (0.58, 0.99)	1.06 (0.79, 1.36)	704 (515, 901)	0.03 (0.02, 0.03)	0.04 (0.03, 0.05)
2021(n, 95%%UI)	56835 (42680, 73440)	1.44 (1.08, 1.85)	1.38 (1.04, 1.79)	2029 (1510, 2596)	0.05 (0.04, 0.07)	0.05 (0.04, 0.07)
Global Women						
1990(n, 95%%UI)	40936 (31059, 52907)	1.55 (1.17, 2)	1.87 (1.42, 2.41)	1494 (1117, 1923)	0.06 (0.04, 0.07)	0.07 (0.05, 0.09)
2021(n, 95%%UI)	88120 (64992, 114469)	2.24 (1.65, 2.91)	1.96 (1.44, 2.55)	3225 (2329, 4164)	0.08 (0.06, 0.11)	0.07 (0.05, 0.09)
China Male						
1990(n, 95%%UI)	2690 (1748, 4106)	0.44 (0.29, 0.68)	0.62 (0.4, 0.95)	88 (57, 134)	0.01 (0.01, 0.02)	0.03 (0.02, 0.04)
2021(n, 95%%UI)	12278 (7558, 17839)	1.69 (1.04, 2.45)	1.25 (0.77, 1.8)	455 (281, 653)	0.06 (0.04, 0.09)	0.05 (0.03, 0.07)
China Women						
1990(n, 95%%UI)	5577 (3671, 7973)	0.98 (0.64, 1.4)	1.21 (0.79, 1.71)	187 (123, 265)	0.03 (0.02, 0.05)	0.04 (0.03, 0.06)
2021(n, 95%%UI)	11407 (7183, 16975)	1.64 (1.03, 2.44)	1.07 (0.67, 1.6)	416 (265, 603)	0.06 (0.04, 0.09)	0.04 (0.02, 0.06)

DALYs, disability-adjusted life-years; n, number; UI, uncertainty interval; ASDR, age-standardized DALY rate; ASMR, age-standardized mortality rate.

Among women, the global DALYs and deaths in 2021 were 88,120 and 3,225, respectively, increases of 115.26% and 115.86% from 1990. The crude DALY and death rates in 2021 were 2.24 and 0.08 per 100,000, increases of 44.52% and 33.33% from 1990. The ASDR in 2021 was 1.96 per 100,000, a 4.81% increase from 1990 (**Table 2**).

In China, the DALYs and deaths among men in 2021 were 12,278 and 455, respectively, increases of 356.43% and 417.05% from 1990. The crude DALY and death rates in 2021 were 1.69 and 0.06 per 100,000, increases of 284.09% and 500.00% from 1990. The ASDR and ASMR in 2021 were 1.25 and 0.05 per 100,000, increases of 101.61% and 66.67% from 1990 (**Table 2**).

Among women in China, the DALYs and deaths in 2021 were 11,407 and 416, respectively, increases of 104.54% and 122.46% from 1990. The crude DALY and death rates in 2021 were 1.64 and 0.06 per 100,000, increases of 67.35% and 100.00% from 1990. The ASDR in 2021 was 1.07 per 100,000, a 11.57% decrease from 1990 (**Table 2**).

**Figure 2:**
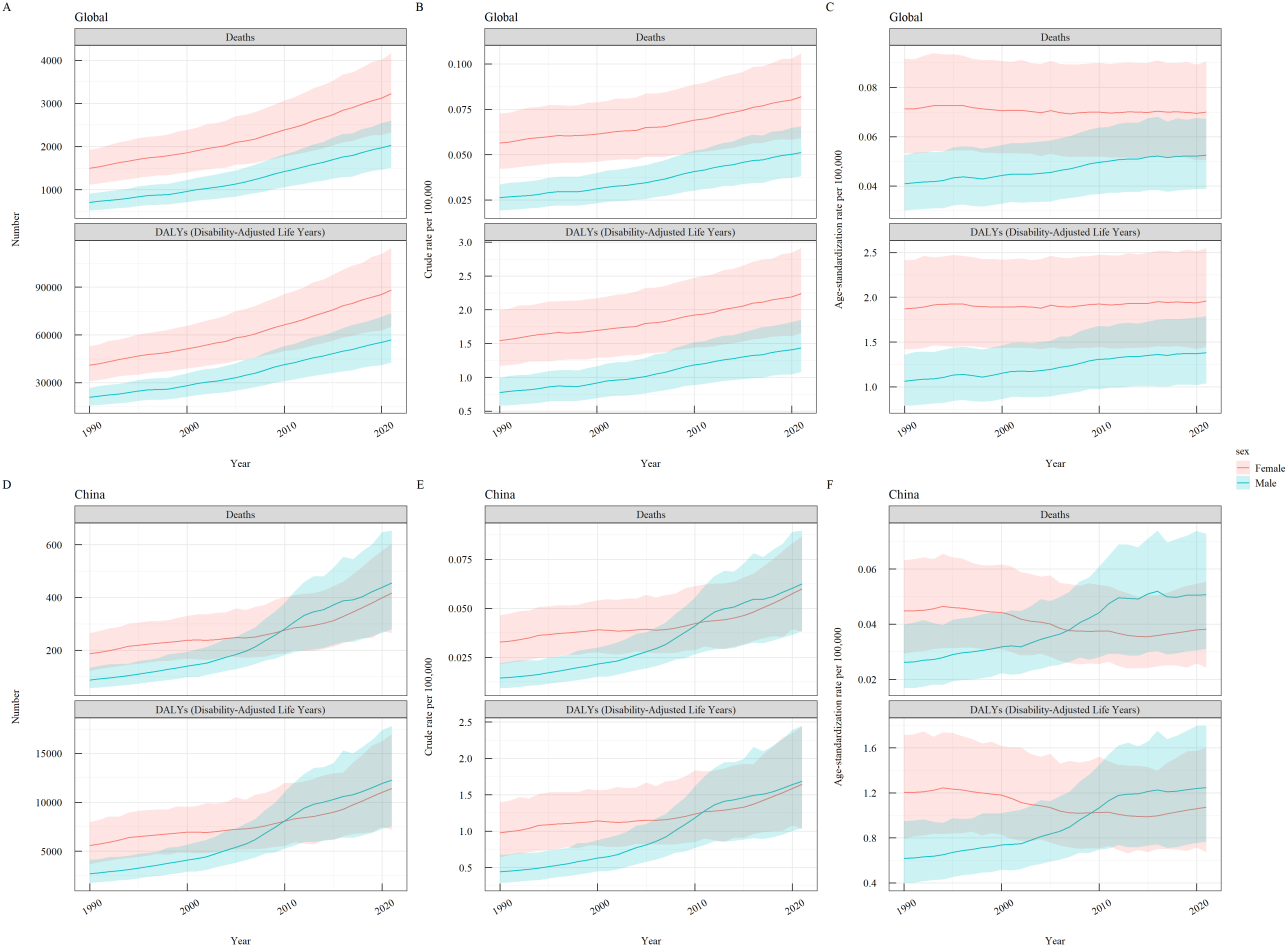
Gender-specific burden of thyroid cancer attributable to high BMI globally and in China, 1990–2021. **(A)** Global Case Numbers; **(B)** Global Crude Rates; **(C)** Global Age-Standardized Rates; **(D)** China Case Numbers; **(E)** China Crude Rates; **(F)** China Age-Standardized Rates.

From 1990 to 2021, the global burden of thyroid cancer attributable to high BMI was higher in women than in men for all indicators (DALY number, death number, crude DALY and Death rates, ASDR, ASMR). In China, the burden was higher in women than in men from 1990 to 2010, but from 2010 to 2021, the burden became higher in men than in women (**Figure 2**).

**Figure 3:**
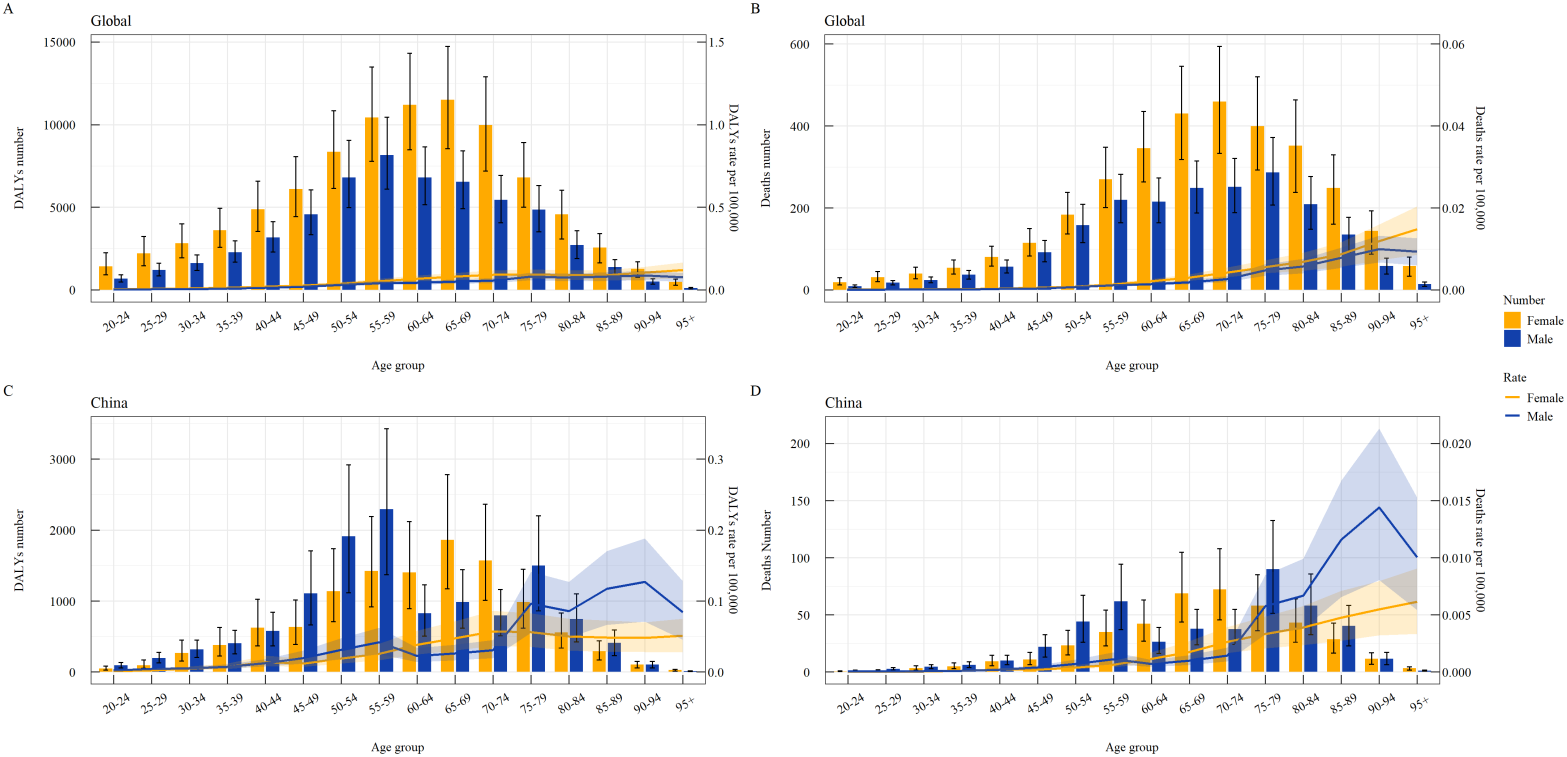
Gender-specific burden of thyroid cancer attributable to high BMI by age group globally and in China in 2021. **(A)** Global DALY Number and Rate; **(B)** Global Death Number and Rate; **(C)** China DALY Number and Rate; **(D)** China Death Number and Rate.

Gender subgroup analysis in 2021 revealed that the DALY number and deaths due to thyroid cancer attributable to high BMI initially increased and then decreased with age. Globally, the peak DALY number occurred in the 65–69 age group for women and the 55–59 age group for men, while the peak deaths occurred in the 70–74 age group for women and the 75–79 age group for men. The DALY and death rates increased with age (**Figures 3A, B**).

In China, the peak DALY number occurred in the 65–69 age group for women and the 55–59 age group for men, while the peak deaths occurred in the 70–74 age group for women and the 75–79 age group for men. The DALY and death rates initially increased and then decreased with age (**Figures 3C, D**).

**Figure 4:**
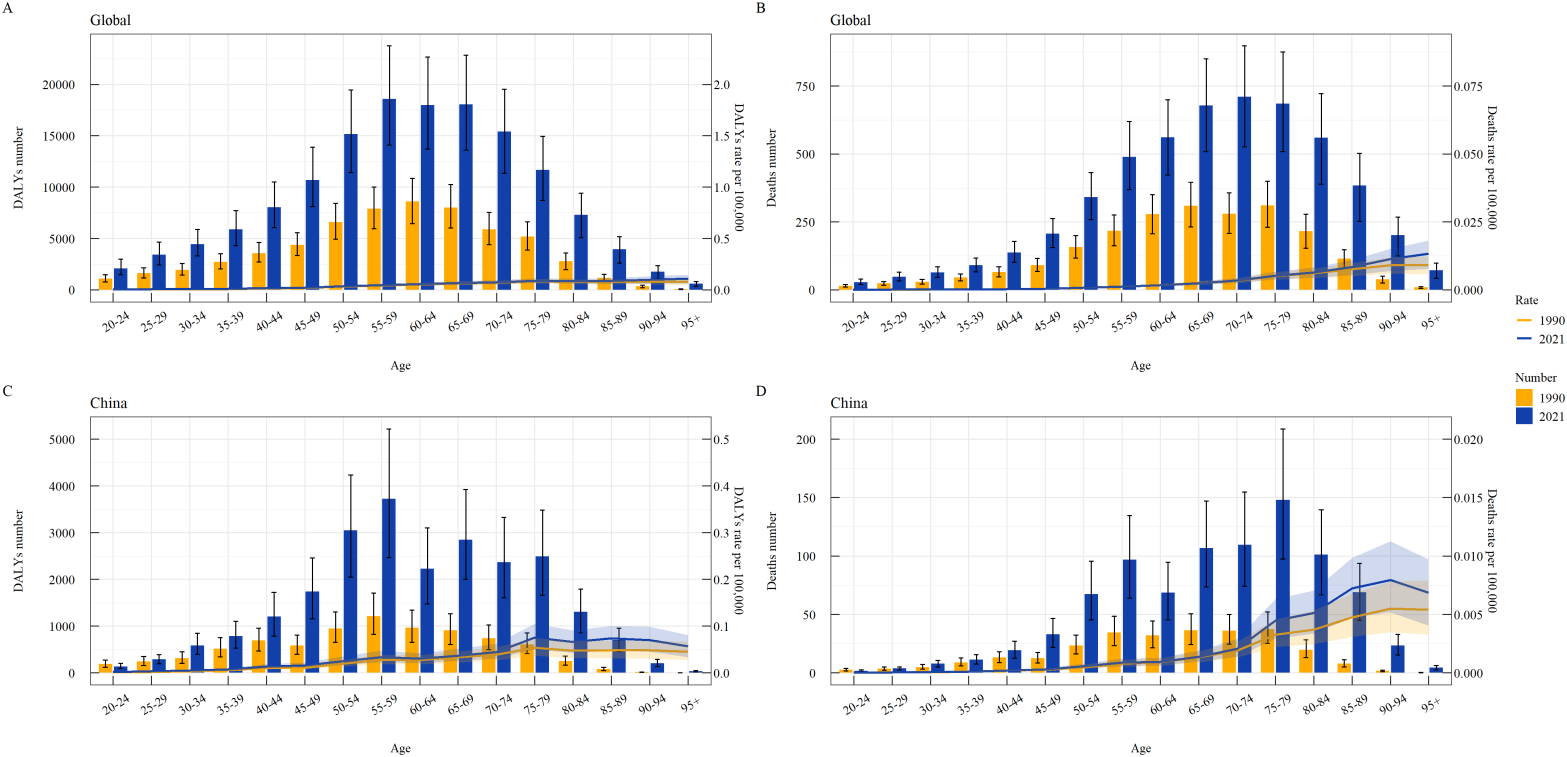
Age-specific burden of thyroid cancer attributable to high BMI globally and in China in 1990 and 2021. **(A)** Global DALY Number and Rate; **(B)** Global Death Number and Rate; **(C)** China DALY Number and Rate; **(D)** China Death Number and Rate.

Age subgroup comparisons between 1990 and 2021 showed that the global DALY number and deaths were concentrated in the 35–84 age group, with DALY and death rates increasing with age (**Figures 4A, B**). In China, the burden was concentrated in the 40–84 age group, with rates initially increasing and then decreasing (**Figures 4C, D**).

### 3.3 Trends in the burden of thyroid cancer attributable to high BMI

**Figure 5:**
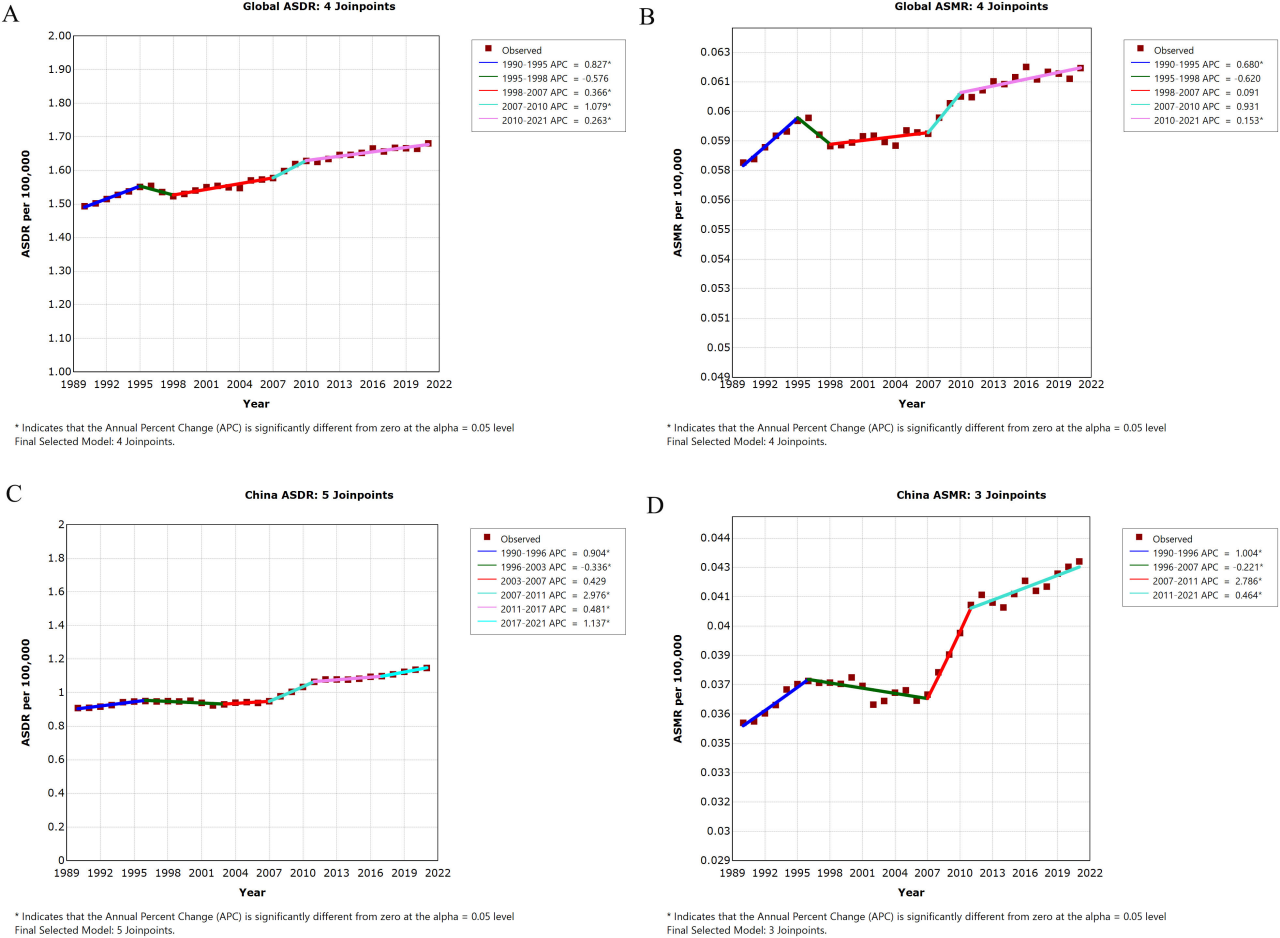
Trends in the burden of thyroid cancer attributable to high BMI globally and in China, 1990–2021. **(A)** Global ASDR; **(B)** Global ASMR; **(C)** China ASDR; **(D)** China ASMR. *:*P<*0.05.

Joinpoint analysis revealed four inflection points in global ASDR (1995, 1998, 2007, 2010), with an AAPC of 0.381 [(95% CI: 0.235, 0.527), *P<*0.05] from 1990 to 2021. The fastest increase occurred from 2007 to 2010 (**Figure 5A**).

Global ASMR showed four inflection points (1995, 1998, 2007, 2010), with an AAPC of 0.220 [(95% CI: 0.071, 0.369), *P<*0.05]. The fastest increase occurred from 1990 to 1995 (**Figure 5B**).

In China, ASDR showed five inflection points (1996, 2003, 2007, 2011, 2017), with an AAPC of 0.773 [(95% CI: 0.591, 0.957), *P<*0.05]. The fastest increase occurred from 2007 to 2011 (**Figure 5C**).

China ASMR showed three inflection points (1996, 2007, 2011), with an AAPC of 0.621 ([95% CI: 0.448, 0.794), *P<*0.05]. The fastest increase occurred from 2007 to 2011 (**Figure 5D**).

### 3.4 Age-period-cohort analysis of thyroid cancer attributable to high BMI, 1992–2021

**Figure 6:**
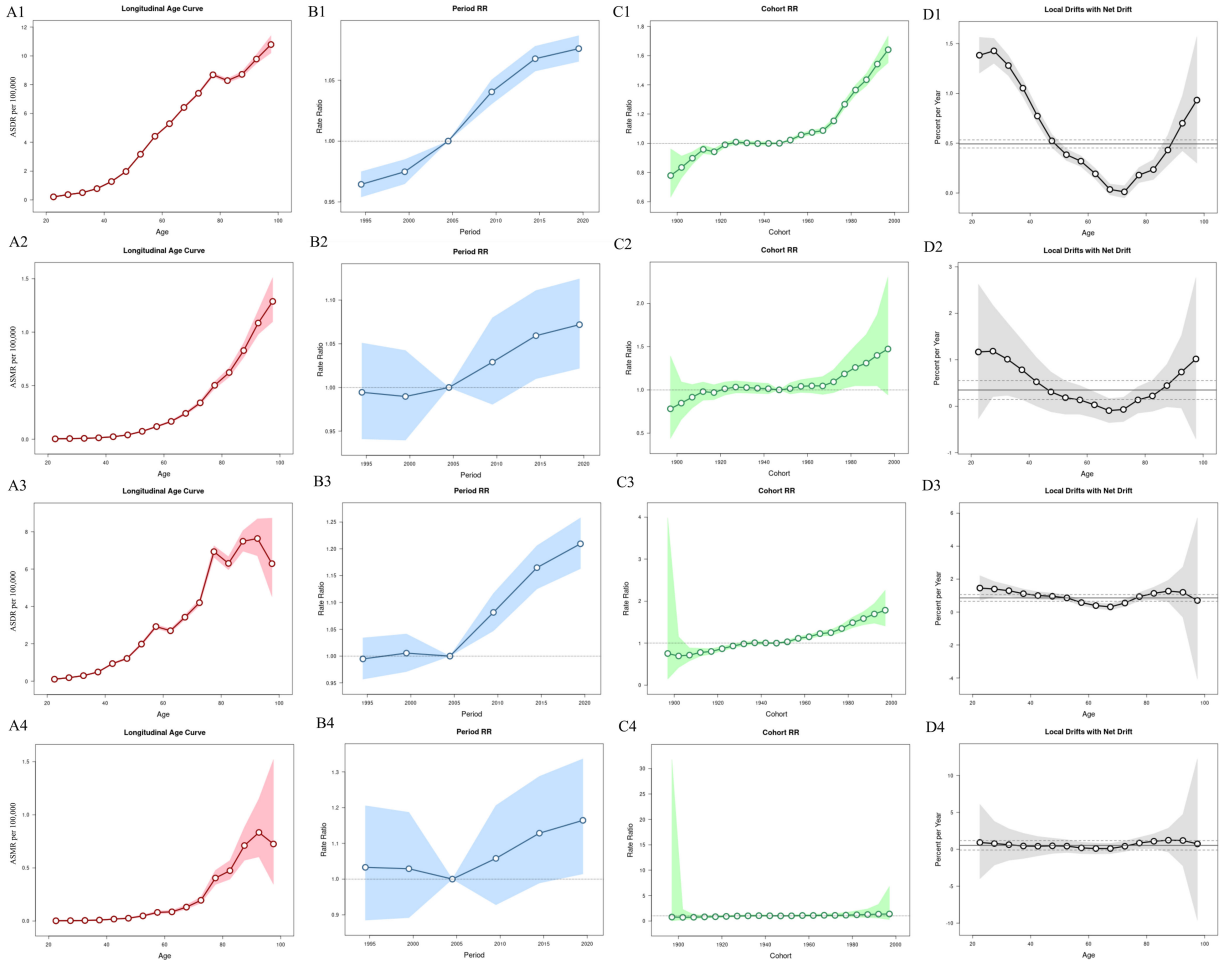
Age-period-cohort analysis of thyroid cancer attributable to high BMI globally and in China, 1992–2021. **(A)** Age Effect; **(B)** Period Effect; **(C)** Cohort Effect; **(D)** Local Drift.

The APC model showed that the burden of thyroid cancer attributable to high BMI was influenced by age, period, and cohort effects. The net drift for the global DALY rate was 0.493% [(95% CI: 0.452%, 0.533%), *P<*0.05], with the age effect showing an increasing trend with age. The period and cohort effects also showed upward trends (**Figure 6**).

For global mortality, the net drift was 0.347% [(95% CI: 0.146%, 0.550%), *P<*0.05], with similar upward trends in age, period, and cohort effects.

In China, the net drift for DALY rate was 0.859% [(95% CI: 0.657%, 1.060%), *P<*0.05], with the age effect showing an initial increase followed by a decline. The period and cohort effects showed upward trends. For mortality, the net drift was 0.534% [(95% CI: -0.100%, 1.173%), *P>*0.05], which was not statistically significant.

### 3.5 Projected burden of thyroid cancer attributable to high BMI, 2022–2031

**Figure 7:**
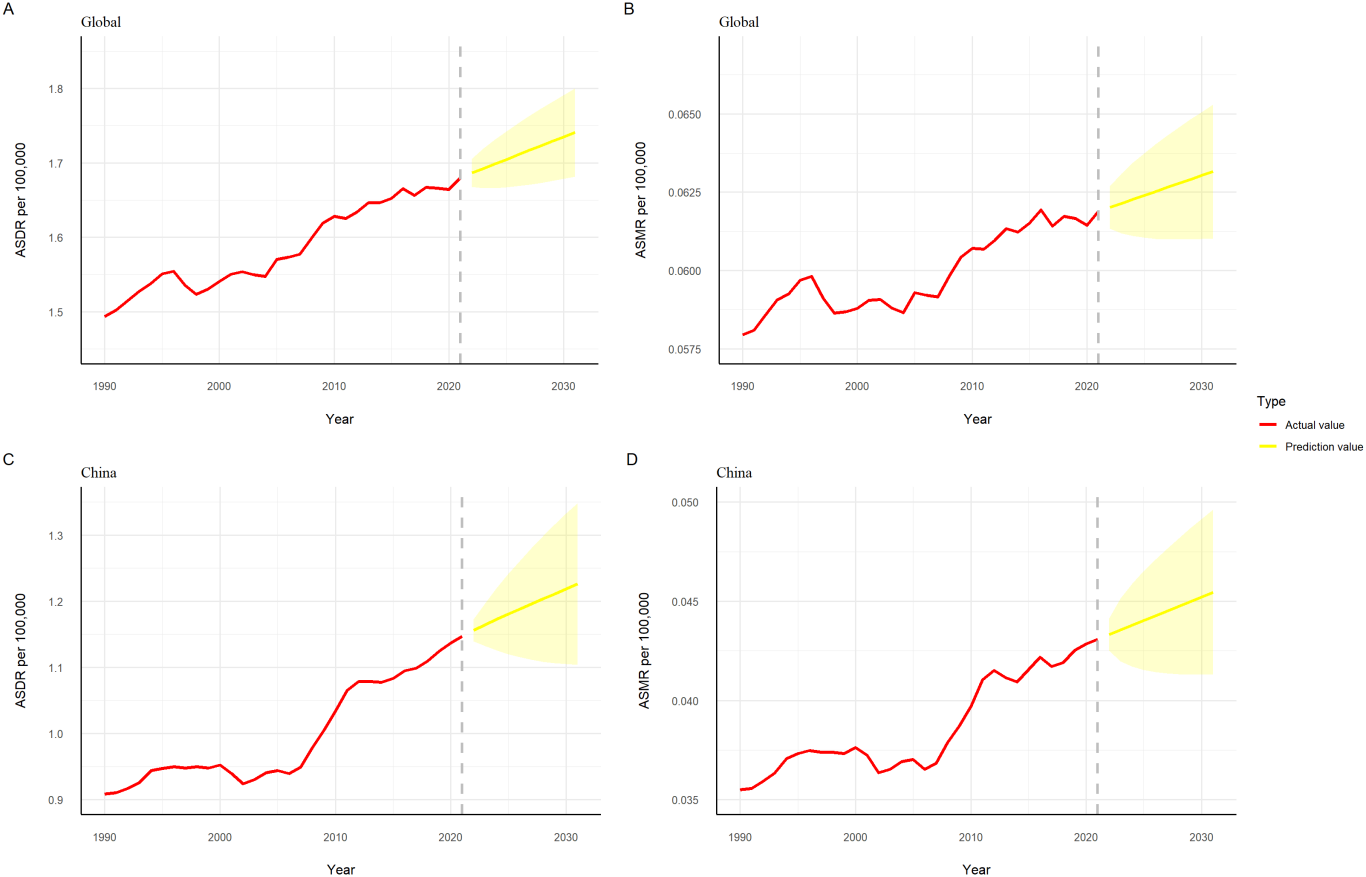
Projected trends in the burden of thyroid cancer attributable to high BMI globally and in China, 2022–2031. **(A)** Global ASDR; **(B)** Global ASMR; **(C)** China ASDR; **(D)** China ASMR.

The ARIMA model predicted that the global and China ASDR and ASMR for thyroid cancer attributable to high BMI would continue to rise from 2022 to 2031 (**Figure 7**).

## 4 Discussion

This study analyzed the trends in the global and China disease burden of thyroid cancer attributable to high BMI from 1990 to 2021. The findings revealed that in 2021, the rates of change in China for DALYs number, crude DALY rate, age-standardized DALY rate (ASDR), deaths number, and crude death rate attributable to high BMI-related thyroid cancer were higher than the corresponding global rates. Over the past three decades, the risk of thyroid cancer attributable to high BMI in China has shown an upward trend. Further subgroup analysis indicated that in 2021, the rates of change for DALYs number, crude DALY rate, ASDR, deaths number, crude death rate, and age-standardized mortality rate (ASMR) among China males were higher than those among males globally. From 1990 to 2021, the global disease burden of thyroid cancer attributable to high BMI was higher in females than in males. In contrast to the global trend, the disease burden among China females was higher than that among males from 1990 to 2010, but this pattern reversed from 2010 to 2021, with males exhibiting a higher burden. This observation aligns with the findings of Meng Z et al. (49, 50). This trend shift may be attributed to biological susceptibility, risk behavior patterns, and variations in health management adherence among (27, 51–54). Metabolic disturbances induced by elevated BMI, including insulin resistance and chronic inflammation, exhibit greater potential to drive malignant progression of thyroid cancer in male populations. The pathophysiological mechanisms may involve BMI-associated alterations in androgen levels, which could further elevate thyroid cancer risk. Notably, male demographic groups demonstrate a higher prevalence of risk-enhancing behaviors such as excessive caloric intake, alcohol consumption, and occupational stress exposure. These factors, combined with the frequent comorbidity of cardiovascular disease and diabetes mellitus in high-BMI males, create a synergistic effect that significantly amplifies thyroid cancer susceptibility (55). Histopathological analyses suggest that male thyroid cancer patients present with higher proportions of poorly differentiated subtypes, which are associated with unfavorable prognosis and consequently contribute to elevated age-standardized disability-adjusted life year rates (ASDR) and mortality rates (ASMR). This epidemiological pattern acquires particular significance in the context of China’s rapidly aging population (56). The expanding male population over 60 years old represents a key risk group. The incidence rate of BMI-related malignancies over 60 years old has reached its peak. Due to the age-related disease burden indicators, the weight in the DALY calculation is too high (57).

Age subgroup analysis revealed that globally in 1990 and 2021, DALY person-years and deaths attributable to high BMI-related thyroid cancer were primarily concentrated in the 35–84 age group, consistent with the findings of HU S et al. (58–60), who reported that thyroid cancer is most common in adults aged 30–60. In China, DALY person-years and deaths were mainly concentrated in the 40–84 age group. These findings highlight the need for tailored screening and treatment strategies for different age groups, particularly for elderly males. Implementing targeted screening strategies, such as adjusting the frequency of ultrasound examinations and adopting more aggressive treatment approaches, is crucial to mitigate the disease burden of thyroid cancer attributable to high BMI.

Joinpoint analysis showed that from 1990 to 2021, the average annual percentage change (APC) in ASDR and ASMR for thyroid cancer attributable to high BMI in China (0.773 and 0.621, respectively) was higher than the corresponding global rates (0.381 and 0.220), indicating a more pronounced upward trend in the disease burden in China during this period. The age effect in the APC model demonstrated that both globally and in China, the DALY rate and mortality rate of thyroid cancer attributable to high BMI increased with age. The period effect revealed an upward trend in DALY risk and mortality risk over time. The cohort effect indicated that DALY risk and mortality risk increased with later birth cohorts. The age effect on the disease burden may be attributed to the following factors: First, metabolic disorders (e.g., insulin resistance and abnormal lipid metabolism) caused by high BMI are more pronounced in older populations (61) and may accelerate thyroid cancer cell proliferation and metastasis in this group (62). Second, the cumulative metabolic damage from long-term high BMI status leads to oxidative stress and reduced DNA repair capacity. With advancing age and prolonged exposure to high BMI, the risk of malignant transformation in thyroid cancer increases, and mortality rises exponentially. The progression of the disease is significantly faster in older individuals compared to younger groups. The period effect may be associated with the cumulative worsening of metabolic abnormalities such as insulin resistance and chronic inflammation caused by high BMI over time. Additionally, the obesity epidemic exhibits a lag effect. The rapid rise in obesity rates in China over the past three decades has resulted in a delayed increase in high BMI-related cancer risks, which become more apparent over time. Furthermore, advancements in medical technology and the widespread use of ultrasound in China after 2010 have influenced the detection rate of thyroid cancer. The cohort effect may be explained by differences in obesity trends among birth cohorts during adolescence and early adulthood. Cohorts born after 1980 were exposed earlier to high-calorie diets and sedentary lifestyles, leading to longer cumulative exposure to high BMI-related metabolic disorders (e.g., insulin resistance and adipokine imbalance), which significantly increases the risk of thyroid cancer in middle age. The cohort born during China’s rapid socioeconomic development (1970–1990) experienced a swift transition from undernutrition to overnutrition, and the interaction between early-life metabolic adaptation mechanisms and later high BMI exposure may exacerbate the progression of thyroid cancer.

The WHO predicts a continued decline in China’s population base in the future. ARIMA model projections suggest that if the current influences of period and cohort factors on thyroid cancer attributable to high BMI persist, the upward trends in ASDR and ASMR are expected to continue over the next decade.

This study has several limitations. First, the accuracy and completeness of GBD estimates may be constrained by variations in data reporting systems and collection methods across countries. Differences in diagnostic standards among countries could also introduce bias into the analysis. Second, due to data limitations, we were unable to explore the epidemiological characteristics of thyroid cancer attributable to high BMI by stage or histological type, including papillary, follicular, medullary, and anaplastic thyroid cancer. Third, the lack of provincial-level data precluded a more detailed analysis of potential factors such as economic, cultural, healthcare resource, and demographic variations in the thyroid cancer burden across different provinces in China.

## 5 Conclusion

In conclusion, in 2021, the rates of change in DALYs number, crude DALY rate, ASDR, deaths number, and crude death rate attributable to high BMI-related thyroid cancer in China were higher than the corresponding global rates. From 1990 to 2021, the global disease burden of thyroid cancer attributable to high BMI was higher in females than in males. In China, the burden was higher in females from 1990 to 2010, but a significant shift occurred from 2010 to 2021, with males surpassing females. The peak burden was observed in middle-aged populations, and the burden increased with age. From 1990 to 2021, the global and China disease burden of thyroid cancer attributable to high BMI showed an upward trend.

## Data Availability

The datasets presented in this study can be found in online repositories. The names of the repository/repositories and accession number(s) can be found below: http://ghdx.healthdata.org/gbd-results-tool.
